# Development and validation of juvenile autoinflammatory disease multidimensional assessment report (JAIMAR)

**DOI:** 10.1186/1546-0096-13-S1-P55

**Published:** 2015-09-28

**Authors:** D Konukbay, D Yildiz, C Acikel, B Sozeri, B Makay, NA Ayaz, K Barut, A Kisaarslan, Y Bilginer, H Peru, O Erdogan, E Unsal, O Kasapcopur, Z Gunduz, A Ravelli, I Kone-Paut, J Frenkel, M Gattorno, S Ozen, E Demirkaya

**Affiliations:** 1 FMF Arthritis Vasculitis and Orphan Disease Research in Paediatric Rheumatology (FAVOR), Ankara, Turkey; 2 Ospedale Gaslini, Genova, Italy; 3 University of Paris Sud, Paris, France; 4 University Medical Center Utrecht, Utrecht, Netherlands

## Introduction

There are lots of effects of auto-inflammatory diseases (e.g. pain, fatigue, fear of attack, lifelong drug use, being nervous and angry, problems at school) and those are quite important to patients but have not been measured with the outcome instruments currently included in clinical trials of auto-inflammatory diseases.

## Objectives

The aim of this study is to develop and validate a new multidimensional questionnaire for assessment of children with auto-inflammatory disease (AID) in standard clinical care.

## Methods

JAIMAR includes 16 parent or patient-centered measures and four dimensions that assess functional status, pain, therapeutic compliance and health-related quality of life (physical, social, school, emotional status) with disease outcome. The JAIMAR is proposed for use as both a proxy-report and a patient self-report, with the suggested age range of 8-18 years for use as a self-report. The study was conducted both children with FMF and their parents in seven different paediatric rheumatology centers from Turkey. To validate the JAIMAR, the Outcome Measures in Rheumatology Clinical Trials (OMERACT) filter for outcome measures in rheumatology was applied.

## Results

The analysis data set was collected between December 2012 - April 2013 from the parents of 250 children with FMF in 351 visits and from 179 children in 187 visits. The median age of the children was 10.64 ± 4.38. The JAIMAR was found to be feasible and to possess face, content, criterion and construct validity. Completing and scoring of the JAIMAR is quick and can be finished approximately in 15 minutes. The Cronbach's alpha coefficient for internal consistency for the JAIMAR dimensions was between 0.507-0.998. Between the test-retest scale scores, there is a significant and a positive correlation from medium level to high level (0.607-0.966). For construct validity all the factor loadings are above 0.30. When the criterion validity is considered, we would say that the correlation level between the each subscale and the related scale spanned from medium (r = 0.329, p<0.0001) to large (r=0.894, p <0.0001). Parents' proxy-reported and children's self-reported data were outstandingly concordant. Cronbach's alpha values were between 0.770-0.989.

## Conclusion

The development of the JAIMAR introduces a new and a multi-dimensional approach in pediatric rheumatology practice. It is a valid tool for children with autoinflammatory disease and will help enhance the quality of care in this group of patients.

